# Progress in Surgery

**Published:** 1896-01-25

**Authors:** 


### PROGRESS IN SURGERY.

### HEAD INJURIES.

Several papers have recently appeared bearing on the mechanism of injuries to the skull and brain. Among them may be cited that of Dr. C. W. Dulles on the "bursting theory" of indirect cranial fractures, and of Mr. Alex. Miles, of Edinburgh,[1] on the diagnostic value of fluid discharges from the ear in head injuries. In this paper Miles expresses doubt as to whether the escape of blood and cerebro-spinal fluid from the ear is characteristic of basal fracture, as is usually taught. That there is a fracture in a large proportion of cases in which these signs are present is undoubted; but he quotes several well-authenticated cases where no such fracture could be detected on the most careful examination.

In considering the subject, four questions are raised: (1) What is the nature and source of the clear fluid? Chemical investigation and physiological knowledge prove that it is undoubtedly cerebro-spinal fluid, and must therefore come from the arachnopial space. (2) What is the source of the blood? This must be either the lacerated vessels of the fractured bone; of the brain membranes, including the venous sinuses; or of the brain tissue itself. Against its coming from the bone are urged (*a*) the copious and long-continued flow; (*b*) the small fissure in the bone; (*c*) the tendency of the blood to coagulate when in contact with a rough surface; (*d*) the fact that similar bleeding does not occur in other fractures. The vessels of the dura mater might account for a good deal of the blood lost, but this blood could only be accompanied by cerebro-spinal fluid if the dura mater in the region of the fracture were completely torn, and this the writer says is extremely rare. Dulles[2] also has observed this. If the blood had its source in a tear of a large venous sinus, compression symptoms rapid and severe, would be expected. It would appear that the arachnopial membrane and the brain tissue itself is the source of the blood which escapes, and, if so, there is no difficulty in understanding the accompanying cerebro-spinal fluid. (3) What is the course taken by these fluids? Keeping in mind the anatomical arrangement of parts, the possibilities seem to be (*a*) through a fissure in the petrous bone, opening directly into the tympanic cavity; (*b*) through a fissure in the petrous bone opening into the internal auditory meatus, spreading into the labyrinth, and thence to the tympanic cavity; (*c*) through a fissure involving the internal auditory meatus, with or without fracture of the labyrinth, but lacerating the sheath of the auditory nerve; or (*d*) along the inside of the sheath of the auditory nerve through a laceration at the point of reflection of the arachnopial on to the nerve trunk, thence through the lamina cribrosa along the branches of the nerve on to the labyrinth, and onwards to the tympanum and outer meatus. In all cases, of course, a rupture of the membrana tympani is a *sine quâ non*. For various reasons, stated at length, Miles believes that the last is the route taken by the fluids. (4) What is the mechanism by which the lesion is produced which permits of the escape of fluids? Various observations and experiments are quoted in support of the view that the disturbance of the cerebro-spinal fluid at the moment of injury so damages the nerve sheaths and their continuations that the fluids can escape. The fracture of the base is a coincidence.

The writer is of opinion that, excluding the extra risks of sepsis, the prognosis is, on the whole, better when bleeding and welling from the ears occur than when they do not.

**The Location of Missiles in and their Removal from the Cranial Cavity.**—Dr. G. R. Fowler, of Brooklyn,[3] contributes an exhaustive paper on this subject. The traumatism of the bullet, he says, is always accompanied by severe concussion. From spent balls concussion is the immediate effect, and in all bullet injuries the incidence is chiefly on the vagus centre, and to a still greater extent on the respiratory centre in the medulla; all other symptoms are obscured by the bulbar signs. Secondary symptoms of gunshot wounds vary very widely, and may even be absent. Various practical points in diagnosis are indicated. (1) In cases where no evidence of perforation exists, the surgeon has to determine whether or not the ball has passed through the bone between a depressed fragment and the adjoining sound bone, the former springing back into its place after the ball has passed. Von Bergmann, König, and Howard have all met such cases. (2) The importance of searching for an exit wound and the close inspection of the bullet, if found, lest only a portion has come out; (3) in cases of shots fired into the mouth the vault of the skull should be searched for bulgings or areas of fracture from within; and (4) when no such condition is found, the nasal fossal and accessory cavities must be examined; or, again, (5) the bullet may glance off the bones at the base and pass into the œsophagus or trachea.

Perforation of the bone being established, the next point to be determined is whether or not the dura mater is pierced, either by the bullet or by splinters from the inner table of the skull. This is best done by removing a trephine circle and then carefully exploring. If the ball is through the dura, then the depth to which it has passed has to be discovered, either by the finger, the bullet probe, or the "telephone probe." The inner table appears to splinter more from spent balls than from those with great velocity. When both tables are broken the inner one suffers most, not, as was formerly supposed, from its greater brittleness, but from the fact that it receives the force of the bullet, plus the force conveyed from the outer to the inner table. In proof of this it is urged that when the vulnerating force is applied from within the outer table is the one which is most extensively splintered. The question of the direction which the projectile may have taken in the brain must always remain uncertain. The position of the firearm at the time of shooting should be carefully considered. It seems doubtful if a ball which has traversed the brain and impinged on the skull will rebound any distance into the brain matter. It is more likely to penetrate the skull. In searching for a missile Fowler uses a ball-tipped slender probe with an indicator, which enables the amount of pressure employed to be determined. Absolute recovery after removal of missiles can only be predicated after a long interval has elapsed, as serious consequences often ensue at a late date. Before proceeding to remove a bullet deeply placed in the brain, the amount of damage which the necessary manipulations will produce must be weighed against the risk of leaving alone. Sepsis and hæmorrhage are the two dangers of operations with this object. In this paper numerous cases are quoted in support of the various propositions.

[1] Edin. Med. Jour., Nov., 1895. [2] N. Y. Med. Jour., July, 1895.

[3] Annals of Surgery, Nov., 1895.

